# The heterogeneity and clonal evolution analysis of the advanced prostate cancer with castration resistance

**DOI:** 10.1186/s12967-023-04320-2

**Published:** 2023-09-19

**Authors:** Ao Liu, Yi Gao, Qi Wang, Wenhao Lin, Zhiyang Ma, Xiaoqun Yang, Lu Chen, Danfeng Xu

**Affiliations:** 1 Present Address: Department of Urology, Ruijin Hospital, Shanghai Jiaotong University School of Medicine, Shanghai, 200025 China; 2 Present Address: Department of Pathology, Ruijin Hospital, Shanghai Jiaotong University School of Medicine, Shanghai, China

**Keywords:** Prostate cancer, Castration resistance, Heterogeneity, Clonal evolution

## Abstract

**Background:**

Nowadays, the incidence rate of advanced and metastatic prostate cancer at the first time of diagnosis grows higher in China yearly. At present, androgen deprivation therapy (ADT) is the primary treatment of advanced prostate cancer. However, after several years of ADT, most patients will ultimately progress to castration-resistant prostate cancer (CRPC). Previous studies mainly focus on Caucasian and very few on East Asian patients.

**Methods:**

In this study, the pre- and post-ADT tumor samples were collected from five Chinese patients with advanced prostate cancer. The whole-exome sequencing, tumor heterogeneity, and clonal evolution pattern were analyzed.

**Results:**

The results showed that the gene mutation pattern and heterogeneity changed significantly after androgen deprivation therapy. Tumor Mutational Burden (TMB) and Copy Number Alteration (CNA) were substantially reduced in the post-treatment group, but the Mutant-allele tumor heterogeneity (MATH), Socio-Demographic Index (SDI), Intratumor heterogeneity (ITH), and weighted Genome Instability Index (wGII) had no significant difference. According to the clone types and characteristics, the presence of main clones in five pre-and post-treatment samples, the clonal evolution pattern can be further classified into two sub-groups (the Homogeneous origin clonal model or the Heterogeneous origin clonal model). The Progression-free survival (PFS) of the patients with the “Homogeneous origin clonal model” was shorter than the “Heterogeneous origin clonal model”. The longer PFS might relate to *MUC7* and *MUC5B* mutations repaired. *ZNF91* mutation might be responsible for resistance to ADT resistance.

**Conclusion:**

Our findings revealed potential genetic regulators to predict the castration resistance and provide insights into the castration resistance processes in advanced prostate cancer. The crosstalk between clonal evolution patterns and tumor microenvironment may also play a role in castration resistance. A multicenter-research including larger populations with different background are needed to confirm our conclusion in the future.

**Supplementary Information:**

The online version contains supplementary material available at 10.1186/s12967-023-04320-2.

## Introduction

Prostate cancer (PCa) is one of the highly prevalent malignant tumors in older men. In Europe and America, the incidence rate of PCa ranks first, and is the second leading cancer leading to male deaths [1]. Although Chinese PCa incidence rate is lower than western countries, in recent years, with the aging of the population, changes in diet structure and lifestyle, and improvement of detection level, more people are diagnosed with PCa in China ever year [2]. Androgen axis plays a critical role in prostate cancer growth and resistance. In recent years, with in-depth research on the resistance mechanism of Castrate-Resistant Prostate Cancer (CRPC), some drugs such as androgen synthesis inhibitor abiraterone and androgen receptor blocker bicalutamide have been successfully used in clinical practice and achieved remarkable results [3]. However, these patients will develop different degrees of resistance after several courses of treatment. Therefore, it is crucial to clarify the detailed mechanism of acquired drug resistance in PCa endocrine therapy, to provide effective clinical treatment plans for patients with endocrine drug resistance and provide new therapeutic targets of androgen-independent PCa.

At present, some research focusing on the mechanism of castration resistance have made some achievements. Many signaling pathways related to androgen resistance, such as Wnt/β-Catenin, and some drug resistance-related gene mutations, such as Androgen Receptor variant 7 (*AR-V7*), have been found [4, 5]. Several studies furthermore found that the drug resistance of prostate cancer is a dynamic process, in which tumor heterogeneity will change [6]. Intra-tumor heterogeneity refers to a variety of cells with different molecular markers and sensitivity to treatment in a single tumor. Intra-tumor heterogeneity contains the different distribution (spatial heterogeneity) of different cell clones or the temporal change (temporal heterogeneity) of cancer cell molecular composition within disease sites [7]. With the development of genomic technology, intra-tumor subclones with different driver mutations were confirmed in breast cancer and renal cell carcinoma. Clone and evolution diversity can be a result of external drug pressure. The selective expansion of pre-existing resistance subpopulations and acquired resistance of residual tumors provide raw materials for tumor cell resistance [8]. Therefore, accurate evaluation of tumor heterogeneity and the level of cell clone evolution is of great significance for predicting the progress of drug resistance in prostate cancer and finding new treatment methods. However, the current drug resistance researches are based mainly on Caucasians, while the data of East Asian patients are relatively rare.

In this study, we collected paired samples of five patients with advanced prostate cancer pre- and post-ADT. We performed exome sequencing to analyze tumor heterogeneity and clonal evolution. We tried to explain whether the resistant cell clones in the clonal evolution model of castration resistance of prostate cancer existed before treatment or evolved after treatment, to infer the origin of the cell line causing drug resistance. This study systematically described the clonal evolution model of CRPC of East Asian origin, providing a basis for the investigation of the drug resistance mechanism of prostate cancer.

## Methods

### Patients

This study was approved by the Ethics Committees of Shanghai Jiao Tong University School of Medicine Affiliated Ruijin Hospital. The pathology database of Ruijin Hospital between 2017 and 2019 was searched, and enrolled patients meet all the following criteria: (a) patients with pathologic diagnosis of prostatic adenocarcinoma, (b) patients undergoing ADT after initial diagnosis, (c) patients clinically diagnosed with CRPC after a period of ADT and other additional treatments, (d) patients undergoing resection or biopsy after progression to the CRPC. Finally, five patients were included and 10 formalin-fixed paraffin-embedded (FFPE) tissue samples were retrieved (2 samples per patient and their blood samples), though the patients received different additional treatments apart from ADT. We used the clinical information in electronic medical records. All the slides were reviewed by two different senior pathologists, and the Gleason score was re-assigned according to the modified Gleason grading system by the 2014 International Society of Urological Pathology (ISUP) consensus conference.

### Whole exome-sequencing

Pre-and post-treatment samples were stored at – 80 ℃, and Leukocytes were isolated from whole blood samples for subsequent DNA extraction. Cell purity was evaluated through the Hematoxylin & Eosin (H&E) staining to screen out low-quality samples (cancer cell composition rate < 50%). DNA was isolated from the FFPE samples using the DNeasy Blood and Tissue Kit (69504, QIAGEN, Venlo, Netherlands). The whole-exome library was prepared from native DNA using the xGen® Exome Research Panel (Integrated DNA Technologies, Inc., Illinois, USA) and the TruePrep DNA Library Prep Kit V2 for Illumina (#TD501, Vazyme, Nanjing, China) following standard protocol. Paired-end DNA sequencing performed on Illumina NovaSeq 6000 platform.

### Somatic mutation, CNV, TMB, and MATH analysis

Pair-end Whole Exome sequencing data read in FastQ format was aligned to the GRCh37 human reference genome using Burrows-Wheeler Aligner (BWA) v.7.17 [8]. Sequencing quality was measured by HsMetrics in Picard (version 2.21.6). Intermediate sorting and deduplication of the Sam/Bam file were performed by sambamba v0.7.1. Recalibration of nonidentical reads was performed by bqsr. Somatic variants and INDEL were called by Strelka2 (version 2.9.10) and Manta with default parameters [9]. Single nucleotide variants (SNV) called by Strelka2 were further filtered by the following criteria: (1) sequencing depth ≥ 20 at variant sites in both control and tumor samples. (2) ≤ 5 alternative reads support the variant in the germline sample. (3) ≥ 5 alternative reads support the variant in the tumor sample Filtered variants were then annotated by Annovar [10] and further filtered by the criteria: (4) < 1% population frequency in all of the following database: Exome Aggregation Consortium (ExAC), ESP6500, the Genome Aggregation Database (gnomAD). Resulting vcf files were converted to Maf format using Vcf2maf for further analysis [11].

Copy number alteration was called from aligned WES data reads using CNA kit (version 0.9.7). Paired tumor samples (pre-and post-treatment) and normal samples (Blood) were analyzed using CNA kit “batch” command. Each input sample is first median-centered, then read-depth bias corrections are performed on each of the samples separately. The burden of copy number alteration was defined as the percentage of regions showing deviated copy numbers among all detected segments as previously described [11].

Weighted Genome Instability Index (WGII) is a method for measuring the percentage of chromosome length with chromosome copy number deviating from the average Ploidy. High wGII indicates a higher proportion of chromosome copy number deviating from ploidy [12].

For the TMB of a tumor sample is calculated by the number of non-synonymous somatic mutations per mega-base in coding regions [13]. Then, TMB burden is defined as: TMB = somatic/L. The tumor Mutant-allele tumor heterogeneity (MATH) score for each patient was calculated following the method described by Mroz and Rocco [14, 15].

### Clonal evolution analysis

Maf format mutation information were read and visualized using R package Maf tools [16]. SNV and CNA information for each sample was passed to PyClone (version 0.13.1) to infer and visualize the subclonal clusters [17]. Only clones containing 2 or more mutations were retained for further analysis the phylogenic evolution relationship among these subclones were constructed by citup (version 0.1.2) and visualized by R package mapscape [18]. Intra-tumor heterogeneity (ITH) was represented by the number of subclones in a sample as previously described. The subclone is calculated by PyClone software. All muations were first clustered based on their vaf with pyclone_binomial algorithm [17]. Shannon diversity index (SDI) was also used to represent the intra-tumor heterogeneity. The index was defined as the entropy of all Pyclone cluster sizes. Drug resistant subclones were defined as subclones existing in both pre-and post-treatment tumor samples. Driver genes were annotated for each mutation in selected clones as previously described. Genes in the driver gene list from reference paper was annotated as driver gene [19]. Functions enrichment for pre-and post-treatment specific genes were performed using R package ClusterProfiler [20].

### Gene ontology (GO) and kyoto encyclopedia of genes and genomes (KEGG)

GO and KEGG enrichment analysis of the mutated genes conducted by ClusterProfiler package in R software [20].

### TCGA data of prostate cancer

We downloaded somatic mutation data of prostate cancer from Genome Data Commons (https://portal.gdc.cancer.gov). Gene mutation information was visualized by the maftools package.

### Statistical analysis

The difference of TMB, CNA burden, ITH, and SDI between pre-and post-treatment patients was compared using Wilcoxon’s rank-sum test.

## Results

### Patient cohort and clinical information

In the Ruijin Hospital Clinical Database, PCa patients with follow-up data were screened. Patients receiving androgen deprivation therapy were included in this study. The primary pathological type was PCa. The histopathology analysis of the patient's pre-and post-treatment samples contained a sufficient number of tumor cells determined by the pathologist. Finally, we collected ten samples from five male patients that met the criteria for total exon sequencing. The patient’s clinical information was shown in Table 1, five patients did not have the same treatment regimen, but all of them used the Hormone Therapy (HT) regimen. The efficacy of this study evaluated using the Response Evaluation Criteria in Solid Tumours (RECIST). Also, of the five patients, 4 had bone metastases, and 1 had pelvic lymph node metastases. Therefore, the rate of bone pain relief is also included in the evaluation system.

**Table1:** Summarized patient’s clinical information and diagnosis parameters

No	Age	Initial PSA (ng/mL)	TPSA (ng/mL)	F/U, time-Mo	Metastasis	Treatment			Evaluation		Bx interval-Mo
						Orchie-ctomy	HT	Chemo-Tx	Initial-T	Post-T	
Pt.1	68	> 100	180	30	lymph node		Yes		V	R	27
Pt.2	65	> 1000	600	14	bone		Yes	Yes	V	R	18
Pt.3	69	< 100	118.3	10	bone	Yes	Yes	Yes	V	R	20
Pt.4	72	> 100	NA	24	bone		Yes	Yes	V	R	20
Pt.5	65	> 100	149	7	bone		Yes		V	R	7

*Pt* patient, *F/U* Follow up, *Mo* months, *HT* hormone therapy, *V* valid, *R* resistance, *Bx* biopsy, *NA* not applicable

### Analysis of gene mutation profiles pre-and post-treatment

**Fig. 1:**
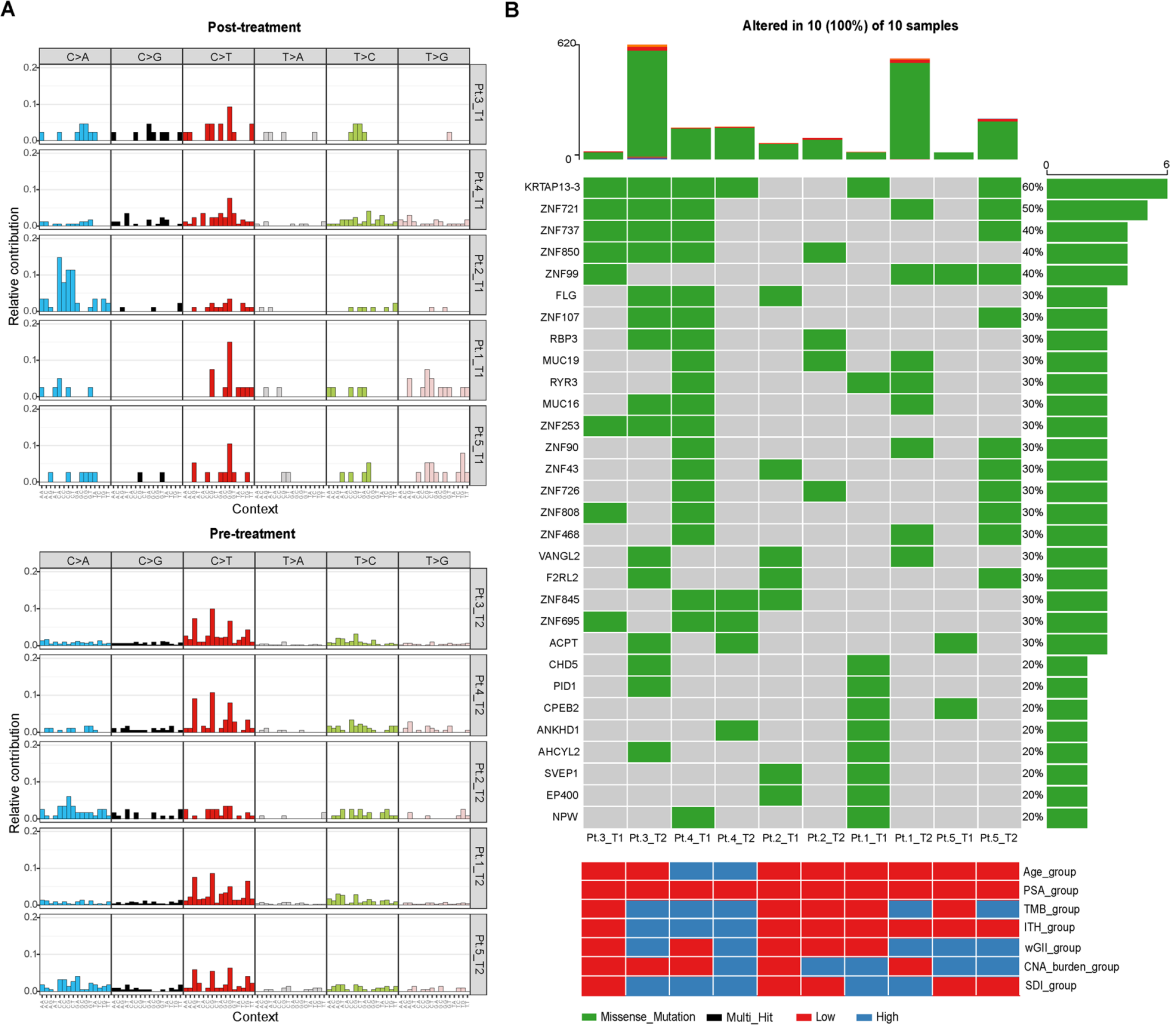
The gene mutation pattern in five prostate cancer patients pre-and post- androgen deprivation therapy. **A** Somatic mutation profiles in 5 prostate cancer patients pre-and post- androgen deprivation therapy; **B** The gene mutation rate in 5 prostate cancer patients pre-and post- androgen deprivation therapy (The cut off of TMB, ITH, CNA, wGII and SDI is their median value, the cutoff of Age is 70, the cutoff of PSA is 10). T1: post- treatment sample; T2: pre-treatment sample; Pt.: patient

With the increase in specific drugs targeting genetic variations, DNA sequencing is becoming a routine management in clinical practice [21, 22]. In this study, Gene Mutation Analysis was performed by exon sequencing in five pre-and post-treatment patients. There was difference between their somatic mutation profiles in the PCa tissue samples from pre- and post-treatment patients (Fig. 1A). Characteristic mutation signature is fingerprints of endogenous and exogenous factors for tumorigenesis, and analysis these mutation signatures may insight for pathogenesis, classification, prognosis, and even treatment decisions [23]. Next, we analyzed the mutational signature of pre-and post-treatment patients (Additional file 1: Fig S1), almost all patients showed the predominant signature changed, except Pt.4 (signature 10). However, the predominant signature change was no similarity and regularity in different patients after treatment. According to Fig. 1B, higher number of gene mutations was observed in the treated samples than in the untreated patients (Additional file 1: Fig S2). Samples from treated patients carry mutations on genes *FLG*, *ZNF43*, *ZNF808*, *ZNF91*, *ZNF347*, *CPEB2*, SEPP*1, EP400,* and *NPW*. Previous studies have shown that androgen deprivation therapy could induce neuroendocrine differentiation of PCa cells [24]. NPW could enhance hormone secretion and regulate germ cell proliferation through adenylate cyclase/protein kinase signal cascade [25]. Therefore, we speculated that *NPW* mutation was probably one of the characteristic genes of castration resistance molecular phenotype. Another gene mutation worthy of special attention, the *EP400* gene, could regulate DNA damage repair through chromatin remodeling function, which played an essential role in the regulation of cell cycle, apoptosis, development, and aging. We speculated that the *EP400* gene might also be one of the key genes relevant to drug-resistant molecular phenotype. Thus, our data suggested that androgen deprivation therapy has a significant effect on the prostate cancer genome, which may be related to drug resistance.

### Analysis of tumor heterogeneity pre- and post-treatment

**Fig. 2:**
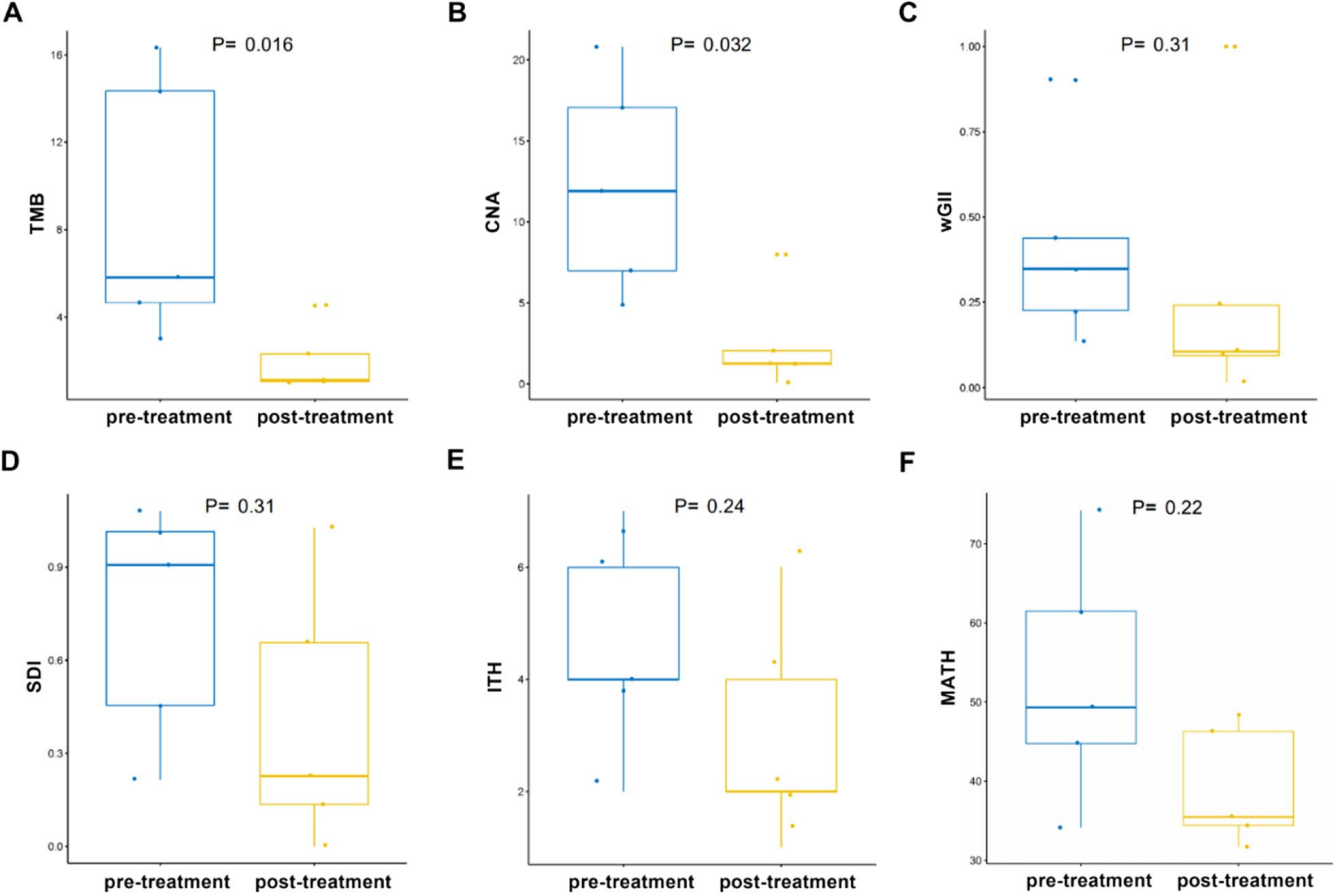
The tumor heterogeneity characteristic of 5 prostate cancer patients pre-and post- androgen deprivation therapy. **A** Distribution of the tumor mutation load; **B** Distribution of CNA load; **C** Distribution of wGII; **D** Distribution of SDI; **E** Distribution of the overall intra tumor heterogeneity estimated; **F** Distribution of the Mutant-allele tumor heterogeneity

Tumor heterogeneity promotes the evolution of tumor, which is a critical obstacle in cancer treatment [26]. Advanced prostate cancer is a highly heterogeneous malignant tumor [27]. Therefore, we further analyzed the effect of drug therapy on the heterogeneity of patient specimens. TMB, CNA, SDI, ITH, WGII and MATH are specific parameters for the evaluation of Heterogeneity. As shown in Table 2 and Fig. 2, the TMB and CNA load of pre-treatment patients are markedly higher than post-treatment patients. In contrast, no significant difference was observed for Sdi, ITH, wGII, and MATH. Low TMB and CNA load, may come from reduction of copy number load. Low TMB with no change in ITH means that although the number of mutations has decreased, tumor heterogeneity may not have changed.

**Table 2:** Summarized patient’s tumor heterogeneity characteristics

	CNA burden		TMB		ITH		SDI		wGII		MATH	
No	Pre	Post	Pre	Post	Pre	Post	Pre	Post	Pre	Post	Pre	Post
Pt.1	6.9911	1.2499	14.3421	1.0526	4	4	0.65637	0.90760	0.242174	0.136519	44.7736	48.4614
Pt.2	4.8805	0.0769	3.02631	2.3158	1	4	0	0.45500	0.105852	0.348067	61.483	35.4696
Pt.3	17.0514	2.0462	16.3157	1.1316	2	7	0.22650	1.01512	0.094325	0.226623	74.2189	31.7062
Pt.4	11.9014	1.2137	4.6579	4.5263	6	6	1.02708	1.08103	0.014809	0.437933	34.1233	34.4248
Pt.5	20.7840	7.9766	5.8158	1.0000	2	2	0.13579	0.21571	0.999692	0.901124	46.2844	49.3191

*pre* pre-treatment, *post* post-treatment

### Pathway enrichment analysis of mutated genes in pre-and post-treatment

**Fig. 3:**
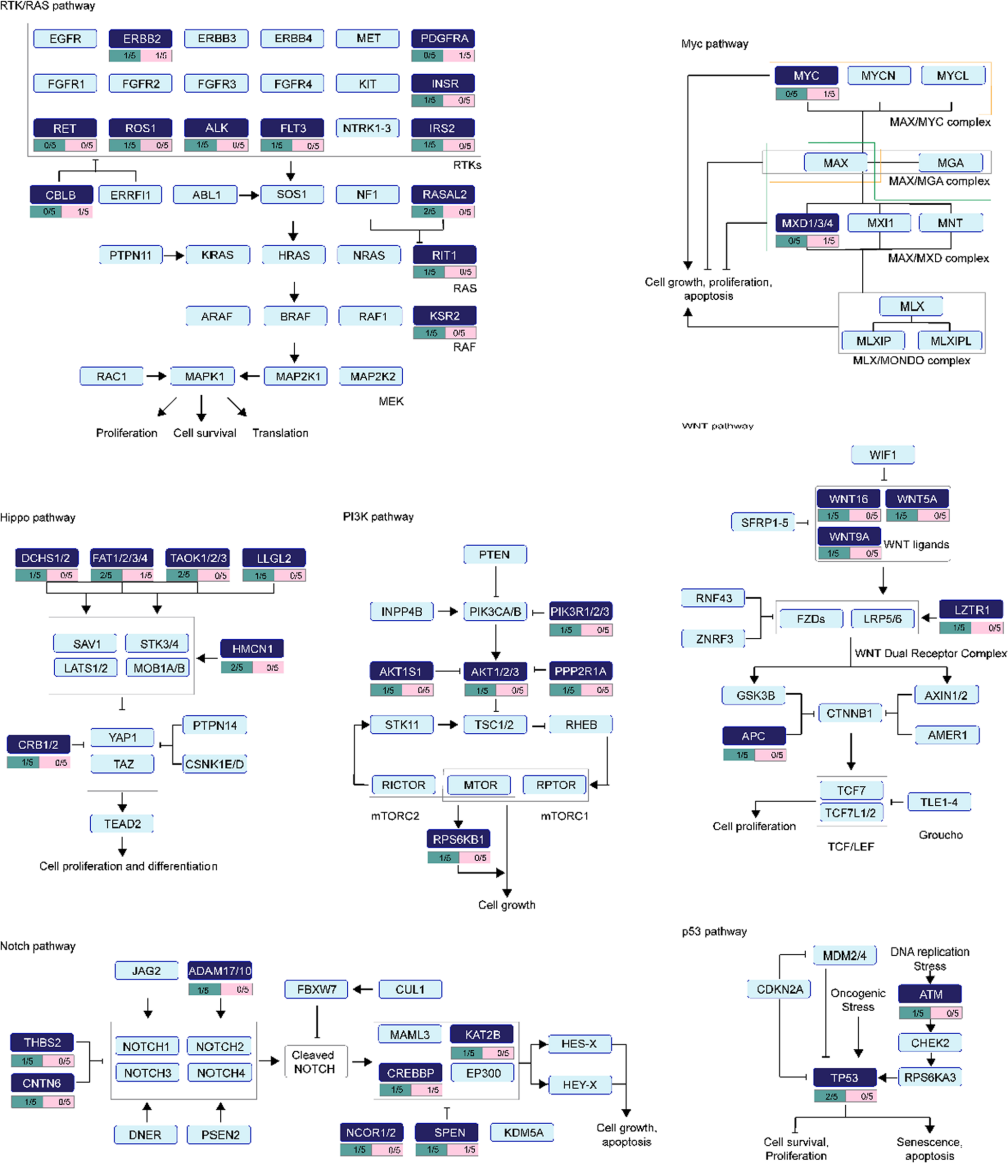
The distribution of the mutation genes in oncogenic signaling pathways pre-and post- androgen deprivation therapy. The deep blue box means the mutation genes detected in the patient samples. The green box means mutation in the pre-treatment; the pink box means mutation in the post-treatment

It has been identified that a mass of genes variation and pathways altered in the disparate cancer types and individual cancer samples [28]. In the current study, 1701 mutations were detected from five pre- and post-treatment patients. We performed the distribution analysis of the mutations in ten typical cancer-related signaling pathways, including TGF-β signaling, Hippo, p53, Nrf2, cell cycle, PI-3-Kinase/Akt, RTK-RAS, Myc, Notch, and β-catenin/Wnt [29]. As shown in Fig. 3, the therapy-related mutations were mainly enriched on seven out of the ten signaling pathways, including the RTK-RAS, Myc, Hippo, PI3K, Wnt, Notch, and p53 signaling pathways. Some of these mutations are on genes involved in drug resistance, such as *ALK*, *CNTN6*, *FAT2*, and *TP53*. And the the number of mutations on these seven signaling pathways decreased after Androgen Deprivation Treatment. This may imply potential therapeutic options. In addition, KEGG and GO enrichment analysis indicated that mutated genes enrichment of patients post-treatment was significantly different from pre-treatment (Additional file 1: Figs S3, S4, S5).

### The Analysis of clonal evolution of pre-and post-treatment PCa samples

**Fig. 4:**
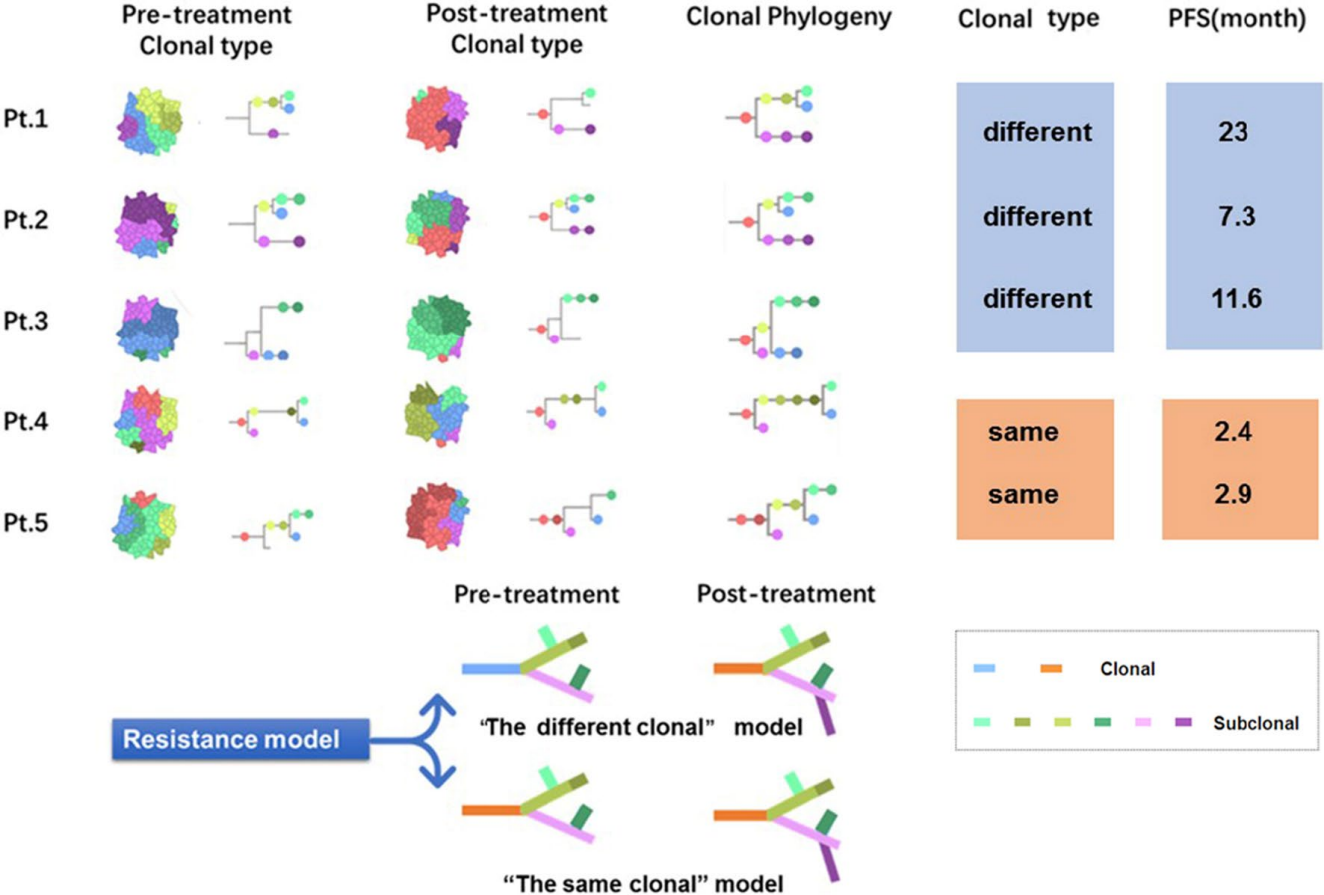
The resistance model of clonal evolution in 5 prostate cancer patients pre-and post- androgen deprivation therapy. *PFS* progression free survival, *Pt.* patient

Recent studies have shown that chemotherapy can fundamentally accelerate the pace of clonal evolution, and lead to new malignant or drug-resistant clones [30, 31]. In this study, clonal/subclonal structure and their evolutionary relationships analysis for the samples from five patients were conducted with Sciclone method. As shown in Fig. 4, the clonal evolution analysis data revealed that all the pre-and post-treatment samples had the same clone family, which indicated that the sample data acquisition was correct. Based on the sub-clone evolution relationship, we found that the five patients' clone evolution patterns were not consistent. In two cases, the main clone was present after treatment as before treatment (the Homogeneous origin clonal model). The progression-free survival time in these two patients was relatively short, at 2.4 and 2.9 months, respectively. In contrast, no primary clone was found in the three other patients after treatment (the Heterogeneous origin clonal model), and their progression-free survival was longer, at 23, 11.6, and 7.3 months, respectively. The results suggest that clonal evolution may be related to the therapeutic effect.

### Effect of gene mutation difference pre- and post-treatment on PFS

**Fig. 5:**
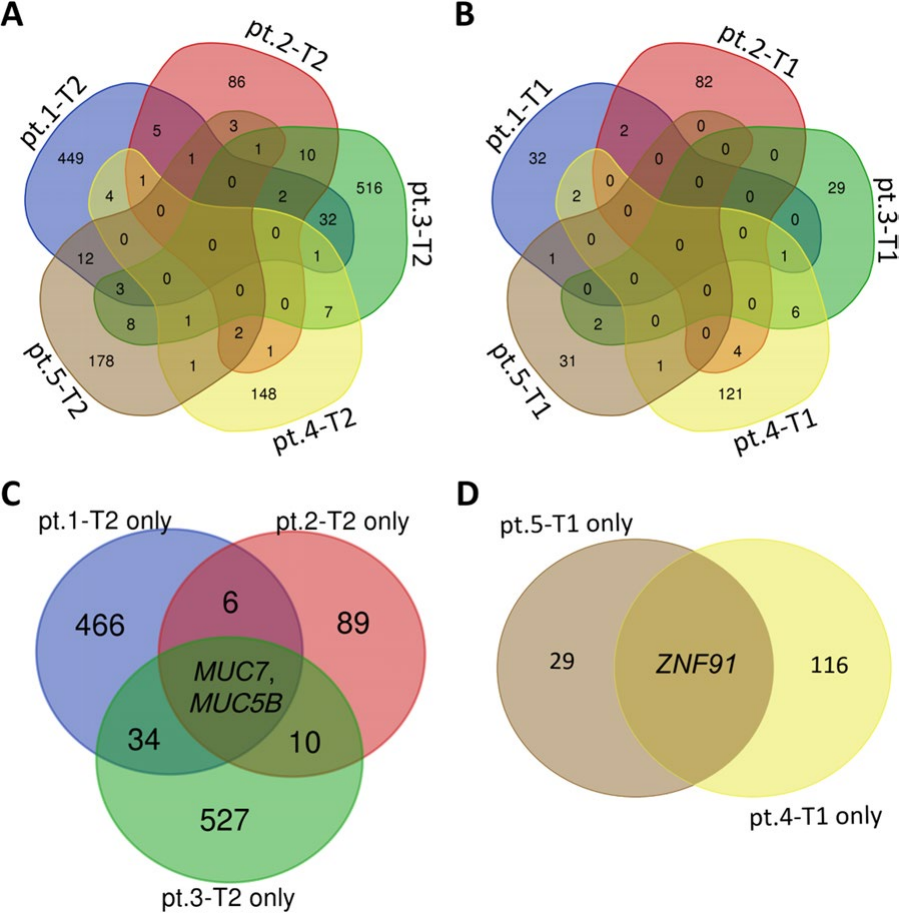
Venn diagram analysis the overlap between the mutation genes in the different patients. **A** Venn diagram analysis revealed no similar genetic mutations before treatment (T2) in five patients. **B** Venn diagram analysis revealed no identical genetic mutations after treatment (T1) in five patients. **C** Only mutation before treatment (T2 only), Venn diagram analysis revealed two same genes (*MUC7* and *MUC5B*) mutated in Pt.1, Pt.2, and Pt.3. **D** The only mutation after treatment (T1 only), Venn diagram analysis revealed only one gene (*ZNF91*) mutated in Pt.4 and Pt.5. Patient: Pt

Each case was characterized by a sizeable number of mutations (Additional file 1: Tables S1, S2). Some of the mutations were repaired after the treatment, while the new mutation genes were created the post-treatment. Before the treatment, none of the five patients had a common gene mutation (Fig. 5A). Moreover, none of the five patients had a common gene mutation after the treatment (Fig. 5B). It is of note that in the Heterogeneous origin clonal model, patients 1, 2, and 3 had two same mutation genes (*MUC7* and *MUC5B*) and that two mutation genes were repaired after the treatment (Fig. 5C). Both *MUC7* and *MUC5B* are members of the mucin family, overexpression and abnormal glycosylation of some transmembrane mucins in adenocarcinoma are associated with aggressive tumor proliferation and poor patient prognosis [32, 33]. We deduced that longer PFS in the Heterogeneous origin clonal model might be related to the repair of *MUC7* and *MUC5B* mutations. Furthermore, in the Homogeneous origin clonal model, patients 4 and 5 had a new mutation of the same gene (*ZNF91*) after treatment (Fig. 5D). ZNF proteins are involved in the regulation of proliferation, apoptosis, cancer pathogenesis, as well as other vital biological activities. The potential role has been revealed to *ZNF91* in some tumorigenesis [34, 35]. Thus, we speculated this emerging mutation may be responsible for resistance to ADT.

### Comparing gene mutation profiles combining TCGA database

**Fig. 6:**
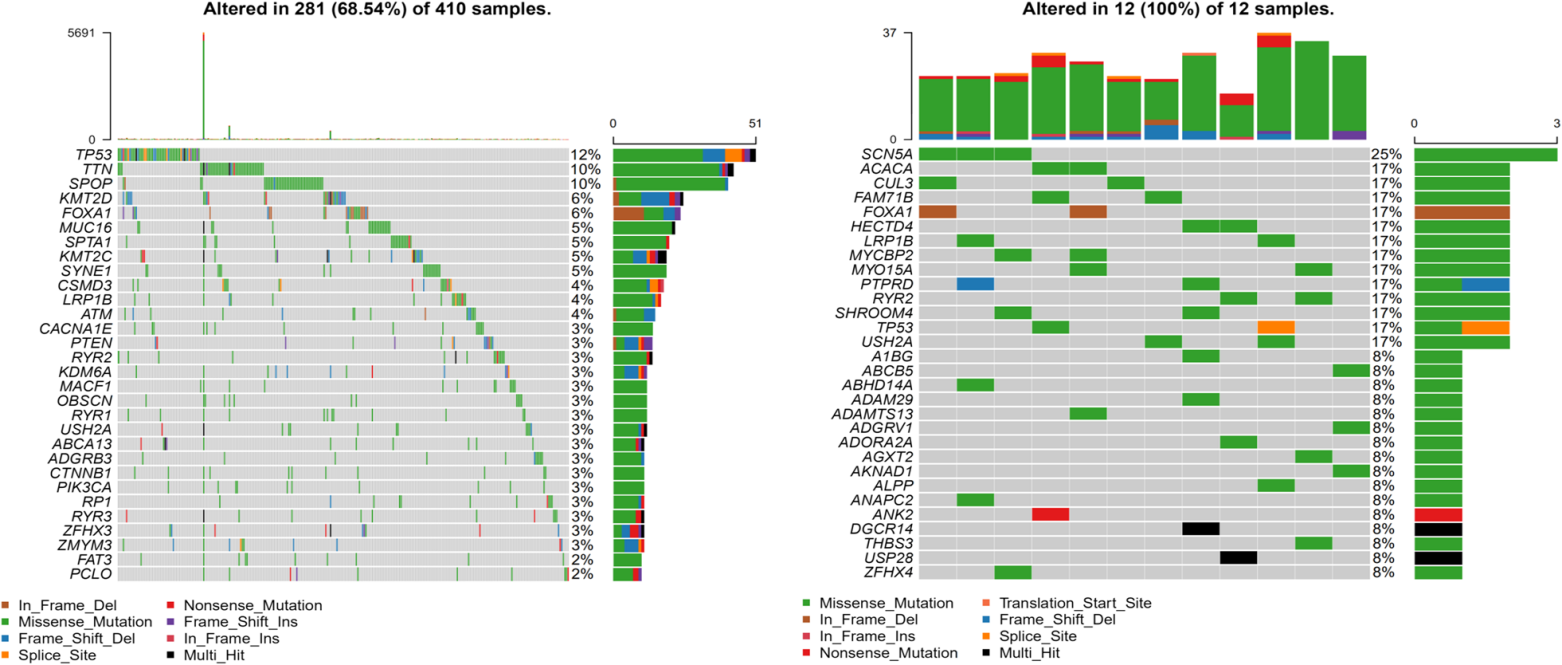
The gene mutation landscape of prostate cancer samples in TCGA datasets. **A** Mutational landscape of Caucasian populations prostate cancer samples in TCGA datasets. **B** Mutational landscape of Asian populations prostate cancer samples in TCGA datasets

**Fig. 7:**
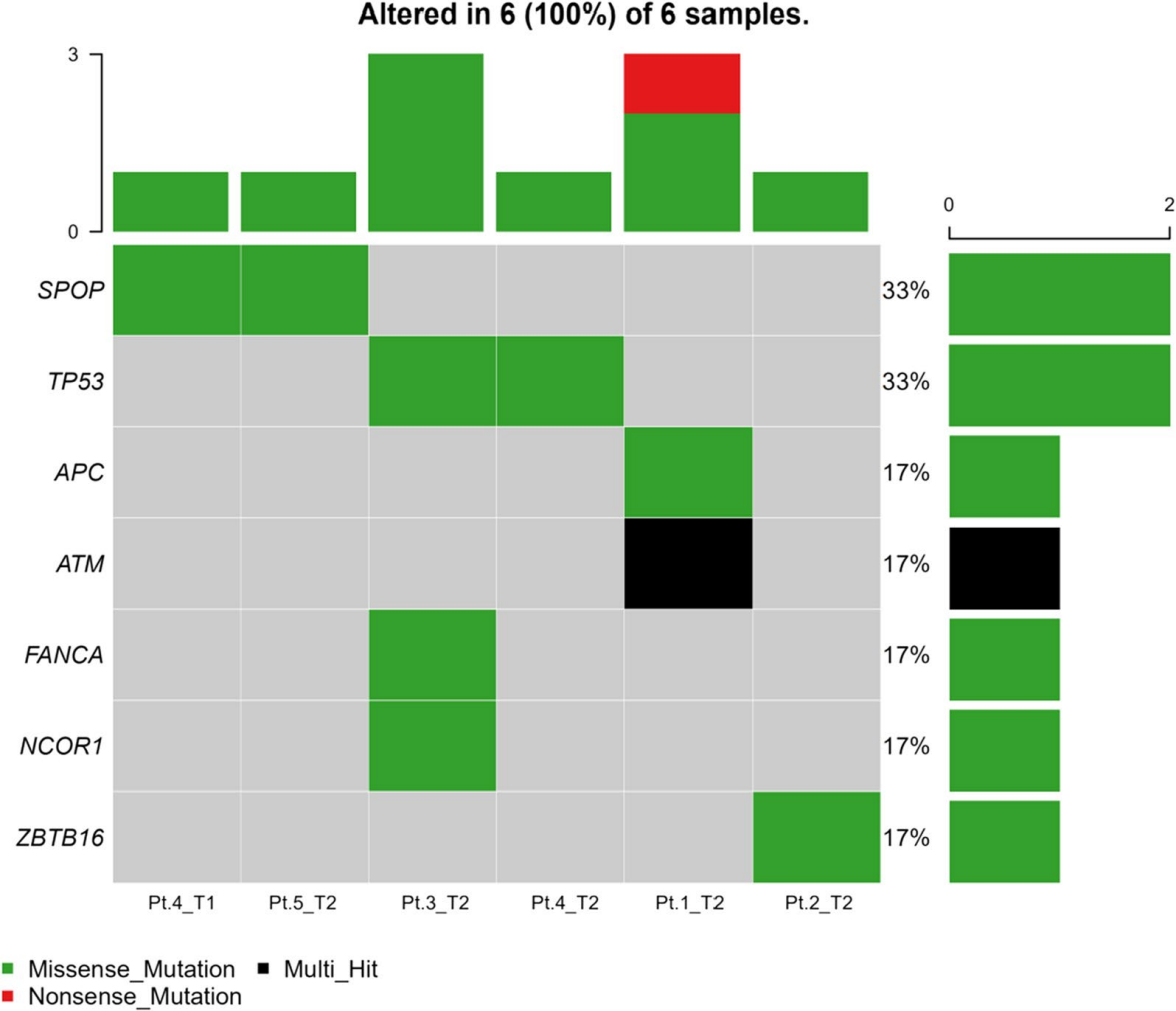
Driver mutation of CPRC samples in our cohort

We analyzed the genetic mutation profiles of Caucasian and Asian prostate cancer patients using information of the TCGA database and found differences between the two populations. In the Caucasian population, the most common aberrant genes in prostate cancer patients were *TP53, TTN, SPOP, KMT2D,* and *FOXA1*, while the common gene mutations in Asian population were *SCN5A, ACACA, CUL3, FAM71B* and *FOXA1*. The top 10 mutation frequency genes were all different except for *FOXA1* (Figs. 6 A and B). In this study, the most frequently aberrant genes were *KRTAP13-3*, *DPP10*, *ALMS1*, *ZNF729*, *ZNF721*, *MUC5B*, *MUC7*, *SYNE2*, *TTN*, *ZNF429*, and *ZNF626* (Fig. 1B). Robinson Dan et al. captured fresh clinical mCRPC biopsy samples in the USA, and found the most common mutation genes in mCRPC were *AR*, *ETS* family genes, and *TP53*. Nearly all the cases harbored at least one cancer-related gene aberration [38]. Similarly, all the cases harbored driver mutation before treatment. Intriguingly, except for Patient 4, none of the other patients harbored driver mutation after treatment (Fig. 7).

## Discussion

At present, the resistant mechanism of PCa development has not been fully elucidated. Previous studies have shown that tumor heterogeneity and clonal evolution may be important reasons for castration resistance of PCa [36]. However, most of the studies were based on whole-genome sequencing of Caucasian patients, but few for East Asian patients. We took advanced PCa patients as the primary research object, then explored the mechanism of acquired drug resistance of PCa endocrine therapy by detecting the gene and molecular phenotype of pre-and post-treatment tumor tissues. Our results suggest that the somatic mutation profile of prostate cancer in East Asian is significantly different from that of Caucasian. The somatic mutations in prostate cancer were significantly altered after treatment with ADT. The significant decrease in CNV as well as TMB may be related to the selection pressure. Additionally, our study initially explored the conjecture of two evolutionary patterns, and demonstrated that patients with heterogeneous evolution have longer PFS. Comparing the mutations in pre- and post- resistant samples, we also fund that MUC7 and MUC5B mutation repair may be associated with longer PFS and ZNF91 mutation may be a key factor contributing to the development of drug resistance in prostate cancer. This study was one of the few studies on the antiandrogen resistance of PCa patients of East Asian origin. Our clonal evolutionary model helps to achieve accurate prediction of PFS in patients with advanced prostate cancer. In addition, the discovery of specific driver genes helps the development of targeted therapeutic agents and promotes the realization of precision medicine treatment in East Asian populations.

By analyzing the genomic data of the pre-and post-treatment samples, we identified 1701 gene mutations. Compared with the pre-treatment samples, we found that after drug treatment, the gene mutation pattern of tumor tissue changed significantly. It was noticed that there were three types of gene mutations in these five samples: 1. Common mutations pre-and post-treatment; 2. Increased mutations after treatment; 3. Mutations disappeared after treatment. Due to the individual differences of patients and different treatment plans, we did not find the mutation gene with identical difference in samples pre- and post-treatment. We found that the mutations of the *NPW* and *EP400* gene had the same pre-and post-treatment mutations in two patients. Our results showed that not all the characteristic genes of drug resistance molecular phenotype appear in the tissues of five patients (pre-and post-treatment). This implies that the acquirement of drug resistance in tumor tissue was a dynamic process, and there was remarkable cell heterogeneity.

This research showed that the development and progression of tumor was a dynamic process resulting into heterogeneity. As a result of this heterogeneity, tumor tissue might include a series of cell populations, which had different genetic characteristics and sensitivity to medical intervention. This heterogeneity might lead to both spatial heterogeneity and temporal heterogeneity of cancer cell molecular composition. Heterogeneity provided fuel for drug resistance [8]. Therefore, accurate evaluation of tumor heterogeneity was essential in the process of finding effective treatment strategies. In this study, we also selected primary indicators to evaluate tumor heterogeneity, including TMB, CNA, SDI, ITH, wGII, etc. to estimate the tumor heterogeneity of these five patients. Compared with the pre-treatment samples, the TMB and CNA of the post-treatment samples decreased significantly, which might be caused by the decrease of copy load. The selective pressure caused by androgen deprivation therapy allowed the resistant clone to change other molecules to preferentially proliferate, leading to the transformation of tumor expression into the androgen-independent type. However, SDI, ITH, and wGII, and MATH did not change significantly. Although antiandrogen therapy affected the complexity of tumor cell variation, it might not affect the heterogeneity in general. Another promising finding was that the results of lower TMB and CNA in resistant tissues suggest that the combination of TMB and CAN may have a more precise predictive signidficance for ADT resistance in PCa, and further studies could group patients according to their TMB and CAN based on expanded sample size to clarify their correlation with drug resistance and the underlying mechanism.

The mechanism of castration resistance also included the change of the AR signaling pathway. The PCa was a kind of hormone-related malignant tumor, and AR played a vital role in its evolution [39]. Mutations of *AR*, splicing variants, up-regulation of *AR* costimulatory factors and down-regulation of suppressor factors, epigenetic changes (DNA methylation and histone modification), and expression of *AR* target gene subtypes mediated by the glucocorticoid receptor (GR) may lead to castration resistance [40, 41]. Besides, the use of other growth and survival factors related to AR signaling axis, like mutations in phosphoinositide 3-kinase (*PI3K*) and *PTEN* [42]. In this study, although there was no classical AR signal pathway enriched in the two data pre-and post-treatment, the mutations of drug resistance-related genes were also identified, such as *PI3K.* Also, epithelial-mesenchymal transition (EMT) referred to the phenomenon in which polar epithelial cells transform their epithelial phenotype into mesenchymal properties with mobility and free movement between cell matrixes. It has been confirmed that EMT was a critical step in early embryonic development, late tissue and organ formation, tissue fibrosis, wound healing, and tumor progression and metastasis. EMT is closely related to aggressive malignant and drug resistance of advanced prostate cancer [43]. EMT is a common phenomenon in prostate cancer, which might be induced by many tiggers (endocrine therapy), and there were interactions among various signaling pathways (Wnt/β-Catenin, TGF-β, EGF, PDGF) [44]. In this study, we identified mutations of Wnt family genes from two pre-and post-treatment data, which suggested that EMT-related drug resistance might occur in these patients [45].

Several studies depicted the genetic landscape of CRPC patients. A comprehensive analysis of prostate cancer genomic in China revealed the genetic disparities between Chinese men and western patients. In Chinese populations, *FOXA1* mutations were the prominent alteration, while *ETS* fusion was regarded as the signature of prostate cancer in western cohorts. There were 18% of Chinese patients had *ZNF292* and *CHD1* deletion. One previous study assessed advanced and metastatic prostate cancer genomic features, and found RB1 loss is significantly associated with poor prognosis, whereas RB1, AR, and TP53 alterations predict shorter PFS in patients with ongoing androgen signaling inhibitors. According to the current datasets, we found that *MUC7* and *MUC5B* mutations repaired was associated with prolonged PFS in CRPC patients. Several studies have reported mucins as predictor of cancers and were related to oncogenesis, invasion and metastasis. *MUC5B* and its evolutionarily related genes *MUC6*, *MUC2*, and *MUC5AC* encode secreted gel-forming mucins. Daniel et al. assessed the expression of mucins in a large colorectal cancer cohort, and found *MUC5B* was positive in nearly a half of cancer patients. Heterogeneity expression of *MUC5B* and other mucins were associated with *BRAF* somatic mutation, tumor location and mismatch repair deficiency. Yang et al. identified *MUC5B* and other cell adhesion related genes as specific signatures of aggressive papillary thyroid microcarcinomas. *MUC5B* has been identified as a signature of invasive mucinous adenocarcinoma and was highly expressed in gastrointestinal, pancreatic, and breast cancer. Yang’s research demonstrated the upstream region of *MUC5B* is bound by transcription factor *SPDEF*, which enhanced the expression of the gene. Our study focused on the association between genetic risks and drug resistance, especially castration resistance. In our dataset, patients with poor prognosis both have *ZNF91* mutation, which indicated *ZNF91* may play a role in drug resistance of CRPC patients. Recently, Whole-exome sequencing was performed in a large series of colorectal cancer patients. *ZNF91* mutation was the one of eight predictors for colorectal cancer and was observed in 14.9% of patients. Here, our dataset demonstrated that *MUC7* and/or *MUC5B* mutations repaired may contribute to longer PFS, and *ZNF91* mutation in post-treatment tissues was involved in drug resistance. We look forward to further laboratory exploration about the underline mechanism of these remarkable mutations.

Clonal evolution patterns were analyzed in samples from pre-and post-treatment patients. We found that five pairs of samples have the Homogeneous origin clonal family, which was in line with the patients’ clinical situation, confirming the reliability of specimens for clonal analysis. However, two different patterns of clone evolution were identified in these patients in the process of clonal evolution map. One advanced the way of transformation, which was the main pre-and post-treatment clone, and the samples after treatment derive their unique subclones based on the same main clone. In the other way of evolution there was no common pre-and post-treatment main clone. The subclones in samples from post-treatment were similar to that of pre-treatment but with new subclones produced. We found a more prolonged progression-free survival in this patient. These results suggested that there was a close relationship between clonal evolution and ADT resistance.

The population and samples in this study has limited physical condition and ethnicity. Our failure to perform multisite biopsies, especially in pre-treatment samples, may have an impact on the diversity of clonal evolutionary analysis. We found a pattern of clonal evolution associated with drug resistance using pre-and post-treatment samples. However, due to the limited sample size, the current results are qualitative rather than quantitative analysis. Therefore, more specimens are needed to determine whether the two evolutionary cloning patterns we found will impact the efficacy of the treatment or not. Additionally, application of computational modeling could also be helpful to validate our finding. Furthermore, the function of drug-resistance-related genes in cloning, especially in co-host cloning, needs further verification, which will be the priority of our next work.

## Conclusion

This study investigated the heterogeneity and clonal evolution of pre-and post-treatment PCa patients. The results reveal the shaping of androgen deprivation therapy to tumor heterogeneity and the selection of clonal evolution patterns. Based on the genomic data of patients, we described for the first time two clonal evolutionary models related to castration resistance of PCa. For the genes determinedd in the resistant clusters, especially those in the co-dominant clones, it will be essential to verify their drug-resistant function in androgen deprivation therapy.

### Supplementary Information

**Additional file 1: Table S1** Mutated gene in patients before treatment. **Table S2** Mutated gene in patients after treatment. **Figure S1.** Cluster dendrogram showing the contribution of each composite mutation signatures to the overall mutation spectrum of each pre- and post-treatment sample. **Figure S2.** The number of mutations for pre-treatment samples (T2) and post-treatment samples (T1). **Figure S3.** Kyoto Encyclopedia of Genes and Genomes (KEGG) enrichment analysis of all mutated genes for pre-treatment samples (T2) and post-treatment samples (T1). **Figure S4.** Gene Ontology (GO) cellular component (CC) enrichment analysis of all mutated genes for pre-treatment samples (T2) and post-treatment samples (T1). **Figure S5.** Gene Ontology (GO) biological process (BP) enrichment analysis of all mutated genes for pre-treatment samples (T2) and post-treatment samples (T1).

## Data Availability

All datasets used and/or analyzed during experiments are available from the corresponding author on reasonable request.
