# Supplementary material for: The heterogeneity and clonal evolution analysis of the advanced prostate cancer with castration resistance
Source: J Transl Med. 2023 Sep 19;21:641. doi: 10.1186/s12967-023-04320-2 (PMC10510184; doi:10.1186/s12967-023-04320-2)
Supplement: Supplementary file 1 — Additional file 1: Table S1 Mutated gene in patients before treatment. Table S2 Mutated gene in patients after treatment. Figure S1. Cluster dendrogram showing the contribution of each composite mutation signatures to the overall mutation spectrum of each pre- and post-treatment sample. Figure S2. The number of mutations for pre-treatment samples (T2) and post-treatment samples (T1). Figure S3. Kyoto Encyclopedia of Genes and Genomes (KEGG) enrichment analysis of all mutated genes for pre-treatment samples (T2) and post-treatment samples (T1). Figure S4. Gene Ontology (GO) cellular component (CC) enrichment analysis of all mutated genes for pre-treatment samples (T2) and post-treatment samples (T1). Figure S5. Gene Ontology (GO) biological process (BP) enrichment analysis of all mutated genes for pre-treatment samples (T2) and post-treatment samples (T1). [file 12967_2023_4320_MOESM1_ESM.docx]

**Table S1 Mutated gene in patients before treatment**

| **Patients** | **Mutation genes** |
| --- | --- |
| **Pt.1-T2** | RYR3, KIF26B, GPR98, NBAS, PANX1, WASL, MACF1, SEMG1, ADCY7, TRIO, C12orf66, ATXN7L1, KIF7, BTN2A1, DHX35, NWD1, SMARCC1, ASCL3, KEL, NUP210L, MFSD6, NLRP3, GNAI2, MYOM1, TMEM19, A2M, UTRN, EPHA2, LRRC8C, ZNF274, RHD, VN1R2, NEK4, BTG1, HIST3H3, ZBTB38, MAGEE1, CMTM1, AQP10, GPR78, BSPH1, USP14, MYO1C, ZNF598, ARNT, HSPBAP1, SLC15A4, PRSS45, UBR3, PAK6, REST, FNDC1, FAM98B, ZSCAN16, INSC, IGSF1, MMP16, C3AR1, SH2B2, ZNF605, RACGAP1, ATP1B4, PPP1R9A, MBTPS1, SECISBP2, TMEM117, CHST11, NEFM, ANKRD28, ADCK4, FYB, USP10, ZZEF1, FAT2, PRKD3, METTL21A, CCDC108, PTPRB, EXOSC10, MAU2, NACAD, WDR6, COL7A1, ITFG2, GLG1, CHGA, SLC35A5, SUN1, ABCC11, ANKLE2, RBM44, DNHD1, ADRA1A, HOXA3, DNAAF1, AFP, DUSP18, FN1, ABCB1, ABCC4, CPNE2, CHMP2B, PCNX, SH2D2A, MEGF10, PCDHGC5, PIK3C2A, F11, TMPO, USP13, NPIPB11, CARD14, TENM1, AHRR, C20orf173, TAS2R46, LEPROT, KIF25, VWA5B1, CYP3A5, KIAA1407, CDADC1, LIMA1, CTSB, CMYA5, TMEM132C, MYOF, AFF4, HNRNPA0, LRRC2, OR10T2, APOA5, HOGA1, ZNF649, ZNF221, TANK, XKR9, SAMD9L, EXOC8, REV3L, NYNRIN, TMPRSS4, KRTAP1-5, PDILT, TPCN2, ROBO1, SPATA22, SPDL1, DOPEY2, LRRN4, EIF5A2, HBD, TEX2, BABAM1, PLEKHA7, MAP1A, CNNM4, OXR1, EFCAB5, NOX4, NT5E, PARP4, DIEXF, HIRIP3, CYP2A7, TAL2, TOX, ATM, SPTBN5, CATSPERD, EQTN, KIAA0232, SGCZ, ADAMTS20, PCED1A, USP45, SCNN1B, IRS2, CSNK2A2, SDK2, XYLT2, TEKT2, MORC1, PTCH1, AMACR, RAB36, PHYH, LARP6, LCE1E, RSPH3, ARID1B, MTUS1, PIEZO1, DIS3, TNFAIP3, KLHDC2, EHBP1, TAAR9, SLC9B2, TMEM41B, M6PR, CORO6, KRTAP1-1, LPHN3, STK39, KANSL1L, SSFA2, CTTNBP2, DHRS9, TNRC6B, WIBG, ZNF33B, EIF4B, EEF1A2, OR51I1, ACE, CCDC102B, HERC1, DCDC1, CCDC19, MST1R, CNTN3, LMO7, HOXD8, ZCCHC6, CLSTN1, SPAG17, KRT9, ANKRD18B, ERAP2, NUP98, DFFA, SERGEF, EXT1, NPHP4, PGK2, SACS, ALOX15B, RALGAPB, CYP4X1, KLHDC7A, CAPN13, C5orf64, PCNXL4, PCDHGB7, TLN2, USP6, IGSF10, ELFN2, CT47B1, TEP1, C6orf163, ZNF229, LRRK1, MSH4, KIF6, CDK5RAP1, OLFM4, NFIX, AAMP, UBL3, TMEM38A, C8B, PTPRF, LRCH2, OR5L2, DMBT1, FAM187B, CHI3L2, NELL1, FLT3, ZNF132, KLHL38, UCP1, MCM7, ATP5G3, AL583828.1, SDK1, SLC16A13, DCLRE1A, ABI1, TRAK2, VGLL2, TAAR5, TGM6, PIF1, MUT, DENND3, TMCO4, RNF144B, AKAP12, MEOX2, ZNF501, AKAP3, TNKS1BP1, CCNT1, CCDC17, ELOVL6, EVI2B, PRKD1, FAM47E-STBD1, ZNF589, ESCO1, FMO5, SCARB2, KLHL18, RAD54L, LILRA1, MKL2, THBS2, PRX, RALGPS2, ARPC1B, BCAS1, DHRS4L2, UBE4B, KIAA0922, NPR2, C4orf51, VSX1, SERAC1, METAP2, FSIP2, WSCD2, JMJD1C, CHRM2, PPRC1, GPRASP1, ZSCAN31, RNF214, FAM194A, DISP2, CNKSR1, KRTAP4-1, COL4A1, SLFN11, UBQLN1, VCAN, B4GALT3, ZDHHC21, GBP3, DNAH8, ROBO2, SHPK, RNF40, ZNF438, RAPGEF5, CDH23, ACMSD, PAXBP1, MAP4K2, FAM71F2, LAMB2, COBL, OR4D5, MTERFD2, SLC38A1, SLC2A4RG, MCM3AP, ZNF431, KIRREL3, TBC1D25, FAM227A, ADAM17, CHRNA3, VANGL1, ZMIZ1, TBRG1, PITRM1, PCDHA8, ANKIB1, PTPRJ, METTL13, COL6A3, SYT14, ABCB11, PCSK5, NDUFA9, PNPLA7, ASXL1, LDLRAD1, XRCC5, AHSG, NNT, SH3RF1, FNDC3B, DLGAP2, MICAL1, ZNF671, MIA3, POP5, OR2F2, ZBTB44, DUS1L, ABLIM3, BSND, APEX2, MMRN1, CD101, ZNF662, DCC, PLVAP, UBE3C, NDUFA10, TMEM247, ZNFX1, FOXO3, APC, ZXDB, TTLL10, TPR, ABCC8, TTLL5, VPS13C, AKAP11, ACOX2, ANXA4, RETSAT, ZNF57, ADAMTSL3, UGT8, ZFP42, RSPH9, ARHGEF10, NUDCD1, USP44, SRRM2, SERPINA5, PPP4R4, GEMIN5, TSPYL6, NPC1, KMT2C, TEX15, PDE4A, RNF6, SAR1B, KAT6B, CENPF, ROS1, FGF23, PCNT, PTCH2, KRT23, ZNF200, OR4F6, CDHR2, AFF3, CTSC |
| **Pt.2-T2** | SLC22A18AS, SOX7, EHF, TOPBP1, KIAA1522, HERC2, EFNB1, KRT1, TCHH, CYP2U1, CPNE4, OGDH, GGA3, FRRS1L, CSTF3, EMILIN1, PRPS2, SPTAN1, LEMD2, ATP13A4, PLXNC1, GORAB, PIK3R3, SLC17A9, OAS1, MFSD2A, MUC2, FURIN, NUGGC, ZBTB16, RAB5A, LRP1B, KLHL35, GUCY2D, SIPA1L3, ELF4, TRMT44, SAFB2, BAG3, FAM69C, POLR3E, EDA2R, NARFL, ZNF681, C1orf131, HCN3, ADCYAP1R1, KIAA0556, CREB3L4, RAN, ACSS3, NPAS2, ENG, PPP1R13L, ARHGAP32, MS4A1, CENPE, DENND2C, SH3TC2, FAM47C, HIST1H2BG, MAGI2, AKAP6, C4orf21, VPS18, LRRC4B, SFTPB, RNPEP, CYP11B1, SLC12A5, ZNF106, EXTL1, GRM7, MAP1LC3A, TRPV3, SAGE1, ZNF28, FAM193B, CACNG6, HMGXB3, PSAPL1, ZNF341, ZNF675, CCT6A, LRIG2, C8orf44 |
| **Pt.3-T2** | MMS19, KANK1, LPP, PTPRCAP, TKT, DCHS2, RGMB, KMT2E, LAMA2, KLK14, FRAS1, COL22A1, LZTR1, NOS2, RBFOX3, RDH13, SLC6A4, FAT4, PAPOLA, C1orf27, KCNK6, STK16, MTNR1B, FBXL6, WSCD1, LRRN2, PAX4, FASN, DIRC2, STK25, FAM129C, AKAP7, RTL1, ACVRL1, PTPN12, ENTPD3, ZNF623, PIK3R5, TGM7, PIGZ, COL1A1, GPR37L1, DACH1, CARD11, SHISA3, PLCG1, SLC2A13, KRT39, ZC3H7A, SIRT6, CCDC141, ZRANB3, LCT, KIAA2018, MS4A2, CNTN4, ALK, DACT3, ANGPT4, GOLGA6L10, CREG2, GJA10, PIWIL3, RIN2, BORA, PRTFDC1, VWA8, GCC2, PLXNA1, DNAH6, OR2T1, AIPL1, TRIM29, OBP2A, TMEM151B, TF, NLRP10, DNAH12, ALG1L, CNOT1, MAGI3, TFR2, DHX33, GCM2, DDX49, GRIK2, UPF3A, IGSF11, ZNF716, MBD5, USP19, ATAD3B, ZNF382, ENDOG, AHCYL2, KRT71, ATF7, KDM4B, LRRC10, LRRC69, BAG1, CACNA1B, TRH, HTR3C, COG3, HIST1H1A, RUNX1, AGTPBP1, GALNT18, PABPC3, CHRNB4, AKR7A3, PIGF, CLDN24, OR2T29, MUC8, OR52E2, FAM175A, OR1D2, MFRP, RNF20, ZBTB43, FAIM3, ITGB7, CYP4A22, DNAJC7, IFITM2, IL1RL2, LTBP2, FAM181B, LRRC66, C2orf78, DZANK1, TIAF1, LAMB3, FMN1, SLC19A3, SLC2A8, MRPS7, C6orf58, OAS3, ANXA6, C22orf42, RDX, AP2A1, CELSR3, SLC34A3, PFKFB1, FBLN1, ADCY3, RP1, COG4, GRIP1, FANCA, LYST, NGFR, MAGEC1, STOML3, CCR2, NAT6, BLM, RIF1, PRUNE, RIMBP2, INVS, SNX13, SAMD8, NGB, DDX60, ZNF518B, PRB2, SIRPG, ZNF578, WNT5A, OPN3, ADAM10, SPEN, ENTPD2, WNT9A, GPR33, REPS2, TTLL2, IMPACT, SETD5, SLC16A2, PRKDC, ZFP90, CRTC2, CLEC4M, PCDH15, NR6A1, PCDHAC2, ZNF750, OR5C1, OR6C76, RITA1, CNTN5, KIR3DL1, NT5C1B, C3orf80, PMFBP1, CDR2L, SDR39U1, FAM89A, ADCY8, GPR182, SLC17A7, SENP3, ORC5, AMER3, ULBP1, MUC4, ABLIM2, SLC9A3R2, AC006547.14, KHDRBS2, MGMT, NUTM2F, BUB1, SMG1, LZTS3, ATG16L2, AP3B2, OR2T11, ARHGAP23, SEH1L, PRB3, KIF5C, GBP4, PKP2, AMPD3, HIST1H4K, KLK15, CHAD, SIGLEC6, TROAP, ZMYND10, WNT16, RPP30, CPT2, HEATR1, PTGER4, MAST1, TOPORS, RHO, FAM13A, FN3K, MS4A3, TBC1D16, ATP6V0A4, OR2T4, CHTOP, SSPO, SEC23A, DSG4, GPR155, CNBD1, SRCIN1, PTPRK, MTUS2, GAPVD1, DIAPH3, KDR, ACSS1, SRCAP, GRB10, DPF3, TRPM7, IKZF2, EXOC4, PCDP1, AGBL3, PLD2, C12orf55, KRT73, ANKMY1, MYOM2, SLC44A3, BACE1, ZNF619, CTNS, KLHL1, AGRN, TBCE, TATDN2, SDCBP, CHD7, C7orf66, KCNS1, TNK1, POLR1B, SH3PXD2B, CLEC14A, YBEY, NFIB, MBD6, PIK3CG, LPA, ZSWIM2, TRIM13, NAT9, C12orf43, ADAMTS15, UNC5C, NDST2, TMEM136, C10orf71, ANKRD36C, CCDC53, EMC10, DCUN1D1, KDM4C, ZSCAN21, USP21, NEO1, AP5B1, ACSM1, PXDNL, FIGNL1, NAPSA, ZHX2, COX18, WDR87, AGL, BAZ2A, SRRM5, PRB1, ANKRD62, PLSCR1, TCERG1, MPP6, ZNF460, SZT2, ANKRD36, MUC17, UBXN11, EHD4, TOP2A, TEKT4, PPIF, WDR72, SCN1B, PID1, GNPDA2, HCFC2, EIF4ENIF1, NCOR1, MAP2K7, SRGAP1, LACC1, TIMMDC1, LRRCC1, SPTA1, CCT3, DCP2, ZNF121, PIWIL2, TTYH2, SYNE1, UVSSA, TEX37, HIF1A, RYR2, TRIM71, WWC3, ADAP1, OIT3, SLC26A9, PKHD1L1, TRIM28, MATN1, TMPRSS11F, FCRL4, C19orf54, BAHD1, DNAH10, LYSMD3, NMRK2, RIT1, ZEB2, XBP1, TBC1D32, SETD1B, ZNF180, AKT1S1, STK35, OR5K2, MICU2, TMX1, ZFYVE16, DNAJB2, MAP9, TDRD6, TKTL2, KCNC4, FAM83F, IFI35, EYS, GBF1, CCR10, CCDC88C, NVL, TMEM123, POTEH, GOLGA6L1, ZNF560, CCDC157, C19orf24, C19orf57, GPR137, PTCHD3, FLG, MDN1, GRIN3B, CTNNAL1, CLEC16A, MARCKSL1, PPIP5K2, ZNF469, MYO1F, SERPINB10, DNAJB12, PCDHA1, TTC6, ATXN1, CHD5, LAMC2, KRTAP4-7, ZNF462, KIAA0226, RIMS2, FRMPD1, TTC14, TSR1, KCNQ3, SOCS5, IFIH1, TBC1D2, CUX1, ANKRD20A4, NFXL1, LRP4, ASTN1, PCTP, ZNF253, ARHGAP35, HAX1, PKD2L1, OR10G4, PREX1, GTF2IRD1, TIMM17B, KRTAP9-4, RSC1A1, ERICH1, ADSS, F12, CNNM3, AGAP1, TBC1D29, FREM1, LAMA4, DOCK3, THSD4, ZNF250, CYB5A, LPIN1, CLEC4F, FAM131B, ZSWIM3, PRPF40B, TMLHE, AHNAK2, RELN, MALSU1, ZSCAN22, CEP112, ERGIC2, NRG1, MARVELD2, HNRNPU, FLII, C20orf96, TMEM88B, OR52L1, ANKS3, AAK1, CRB1, CYP24A1, ZC3H12B, ANO6, GSTT1, COL6A6, DNAH5, SLC18B1, ICAM5, ZNF678, ARID1A, SEMA3C, PPM1D, KAT2B, OR6C3, COL12A1, ZNF358, ZNF17, XYLT1, ADAMTS7, GOLPH3L, ALPK2, SPOCD1, PDE3B, PMS2, RPS6KB1, ABHD1, LZTS2, PLXNA3 |
| **Pt.4-T2** | CREB3L1, OR6X1, FAN1, STYK1, DNAI1, COL20A1, CYLC2, ST6GALNAC5, DIP2A, KIAA1210, PTPRZ1, TOP3B, LRGUK, BIRC5, OR10J5, SCUBE3, PCDHA13, PGBD5, FRS2, EMC1, RBMXL3, PANX3, MGAT1, LLGL2, USP7, NOVA2, SNX19, SEC23IP, B3GALT5, CRYBA4, KRT37, PNMAL2, RET, C6orf183, DYM, CPAMD8, DISC1, SLC22A8, EMID1, CEBPD, ITSN1, DIXDC1, PSD2, PAWR, ELN, BAI1, PDK2, SLC7A4, TRAPPC5, ZBTB26, LHX8, KDM6B, VPS37D, FKBP5, OBSCN, PDE3A, ENPP2, VAV2, VPS13A, SGSM3, MAPK8IP2, CHPF2, TADA1, AZU1, MYO7B, ADCY10, GABRA1, NRROS, RECQL5, ACOX3, ZFR2, RNF213, ZNF461, SLC7A2, NANOGNB, ZNF695, FAM20B, RNF207, MAN1A1, KCNQ4, DUSP6, KRTCAP3, ZNF430, KRT4, LRRC23, CPXM1, NCAM2, SLITRK5, SH3BP5, ERBB2, HOMER1, SERPINA11, ANKHD1, RNF148, FAM154A, KIAA1549, SHROOM3, AKR1B1, FAH, PLA2G7, NEURL1, TRRAP, LRWD1, EDEM1, C17orf98, SUSD4, KIAA1731, PTPRA, CCDC144NL, MRGPRE, SALL4, NKX1-2, KCTD19, CFHR2, SGSH, LPPR1, PLEKHD1, LRRFIP2, IQSEC3, UMOD, DNAJC17, NPY2R, PABPC1, ANKRD30A, DPY19L4, EGLN3, SEMA4A, KSR2, ZNF789, AMT, IGFBP3, WDR52, CEACAM1, TBCD, MORF4L1, ANKRD13D, KRT85, PCNXL2, ZNF845, GAREML, TEX101, DUSP9, TMC7, ATP10B, CTC1, VEGFB, C1QTNF8, PSD3 |
| **Pt.5-T2** | UTP20, HPN, RBM15, BIVM-ERCC5, RFX5, TMCC3, ZNF208, MCRS1, MPPE1, HPS6, MAN2A2, DEFB116, ZNF43, DPP4, AVPR1B, CORO2A, NDUFA8, THBS3, COPA, ZNF239, POLM, MAEL, ADAMTS16, PDCL2, ZBTB11, ZNF334, LRRK2, ZNF549, DSG2, LPXN, ZNF407, AHR, ASH1L, KLF6, UBQLN2, MYOD1, ACOT2, PPM1M, ZNF891, YWHAH, ZFHX4, STOM, CD248, ABCC12, CREBBP, SLC1A2, MYOZ2, FAM120A, PPOX, PHF19, SPP1, VPS9D1, SMCO2, VTN, SLC35G1, SLC17A4, HTRA3, TECPR2, FAM170B, CAND2, PRUNE2, AMIGO3, TRIM14, ATG9A, PUS7L, E2F7, MORN5, ARID2, MRPS30, CD86, ABHD17C, EFCAB3, POPDC2, TARBP1, DPP6, PRDM1, DNAH2, C16orf78, SH2D4A, HRNR, URB2, TBC1D24, FAM111A, PDZK1IP1, KCNK2, WDTC1, SMYD4, FAM126A, OCEL1, RB1CC1, KMT2D, BTN1A1, PSAP, OR2A12, CCDC3, CEP290, ANXA10, USP31, LRRC24, MTA3, AASS, ZNF16, SPOP, AKT2, ZNF808, MUC6, DZIP1, SLC25A17, PLCL1, CAD, ZDHHC20, TENM3, PPP2R1A, LRRC70, KLF17, AKAP9, DNAH9, C20orf26, LRRC40, PLEC, RANBP17, TPO, CCBL1, UNC13A, TRIOBP, ZFYVE26, POLR3H, DDX17, ZNF100, FAM84A, PEG3, YLPM1, ZNF185, EIF2AK1, KANK2, ZNF594, INSR, DZIP1L, INTS4, CDCA2, SPINK6, CIT, IER3IP1, ILKAP, TANGO6, PTPN3, ZNF668, RP1L1, MLKL, DLC1, PAPLN, EFCAB13, ZFYVE27, METTL11B, ATRNL1, CPT1C, TXN2, COL11A1, CASP2, NAA11, TMTC1, LPHN2, BMP5, NCAPG, GJB4, MAP3K19, GLOD5, CNTN6, C9orf62, DPYSL3, APP, VGLL4, UBXN2A, HYAL1, GBX1, MCTP1, ITPR3, ZNF638 |

T2: Pre-treatment; Pt.: Patient.

| **Patients** | **Mutated genes** |
| --- | --- |
| **Pt.1-T1** | TMEM108, PODNL1, CTR9, CXorf23, TMEM158, CCDC132, CHD5, CST9L, PTK7, RADIL, NUAK2, PTPRF, C11orf35, PPP1R14C, ADAT2, STRN3, ANKHD1, MIEF2, STK31, RRP1B, RCSD1, MME, CAPN7, PID1, ZNF467, AHCYL2, C2CD2L, RANBP9, FOXA3, ZNRF1, CANT1, MAGEF1 |
| **Pt.2-T1** | B3GNT5, GPR98, PPM1H, NDUFV3, EHF, SLC22A11, RLBP1, TRIO, SPAG17, MYOF, SPATA31E1, VANGL2, MUC2, PCDH12, KLHL10, F2RL2, ZNF248, EZH1, CREBBP, SLC12A8, RBM26, PLCG2, ATG2B, BSN, SHANK1, SPATA6, CTB-133G6.1, MXD1, PCDH20, ANK3, PPP2R2C, KIAA2018, DUS1L, GGA3, ROR2, FBXW5, TLL1, TSTD2, VIPR1, GAS2L1, ZNF579, TYSND1, BDKRB2, CSMD1, RSRC1, CD163L1, UXT, GLI3, CNOT1, STIM1, SHANK3, PDIA4, GEM, ZZEF1, THBS1, ITPRIP, MYLPF, ANK1, FAM132B, SP4, DPP10, NTN3, EXTL3, CABP2, CCT6A, RUFY2, SERPINA9, HSPA4L, PSME4, THEMIS, KTI12, ERCC2, TRPM4, IL17RE, DLEC1, CAPN5, KCNA4, ZNF169, USP9X, TBATA, ESM1, OPA1 |
| **Pt.3-T1** | BMP6, PBX4, CBLB, CNGB1, TM9SF1, EXOC4, SEC16A, TET3, PHLPP2, ZNF493, COX11, UTRN, CYP4F12, HSD11B1, CACNA2D4, GNAQ, HOOK2, C11orf65, C2orf80, TOP2A, JUN, RYR1, SHISA9, DIDO1, C1QL4, AVL9, KLHL24, WLS, EEF1A2 |
| **Pt.4-T1** | CREB3L1, RIT2, ZNF727, SPEG, GJC2, NANOGNB, ZNF416, MFSD6L, GAPVD1, ZNF502, ZNF679, CDC27, MKI67, CHAF1A, TDRD10, ZNF107, MUC19, RP1, ZNF93, PCDH1, ZNF586, NFASC, ZNF726, BAI1, DUOXA1, ZNF506, MAP3K5, CYB561D1, PDGFRA, SART3, HRK, ANKRD30A, ZNF229, TCHH, SCN8A, SHQ1, PEX26, BIRC5, SPEN, SPOP, LUZP4, NBEA, KRT13, C2CD3, RBP3, ZNF141, NPIPA1, ZNF789, ZNF468, ZNF714, MUC16, KDM6B, ARHGAP12, UBE3A, WDR78, C9orf37, FLG2, HOMER1, LEPRE1, ZNF30, RBMXL3, SERPINA11, ALMS1, FOXN4, EFCAB8, SFI1, SNX19, ZNF117, ZNF544, XRCC1, MUC17, ZNF665, CRYBB1, ZNF331, AK2, ZNF530, MYC, NLGN1, SFSWAP, PIH1D1, ZNF256, ZBTB49, ZNF728, ZNF549, LRRCC1, OTUD6A, CLSTN2, ZNF675, GAREML, ZNF214, AP3B2, F8, LAMA5, ABHD17C, TBXA2R, ZNF813, TCIRG1, PRKRIP1, ZNF320, ZNF227, ZNF678, ZNF90, TRPV5, ZNF527, CORO6, BTG3, ZNF432, C8orf44, LONRF2, FAT3, ZNF540, MMP24, AHNAK, ABCC4, TNFSF12-TNFSF13, ZNF583, CACNA2D3, ZNF33B, ZNF730, EIF2D, ZNF708 |
| **Pt.5-T1** | ABCA3, GIPC3, KIAA1324L, YWHAH, CELSR3, DZIP1L, HOXC13, NPHP4, ATP8B4, GPR179, UTF1, BZRAP1, TAS2R5, FGF11, CUL4A, IGBP1, TJP2, ERBB2, CPT1C, LRRC57, MYH9, NPAT, ACPT, CTNNA3, KIAA0922, TTC18, ZNF853, PJA1, KLF14, HLX, NRXN2 |

**Table S2 Mutated gene in patients after treatment**

T1: Post-treatment; Pt.: Patient.

**
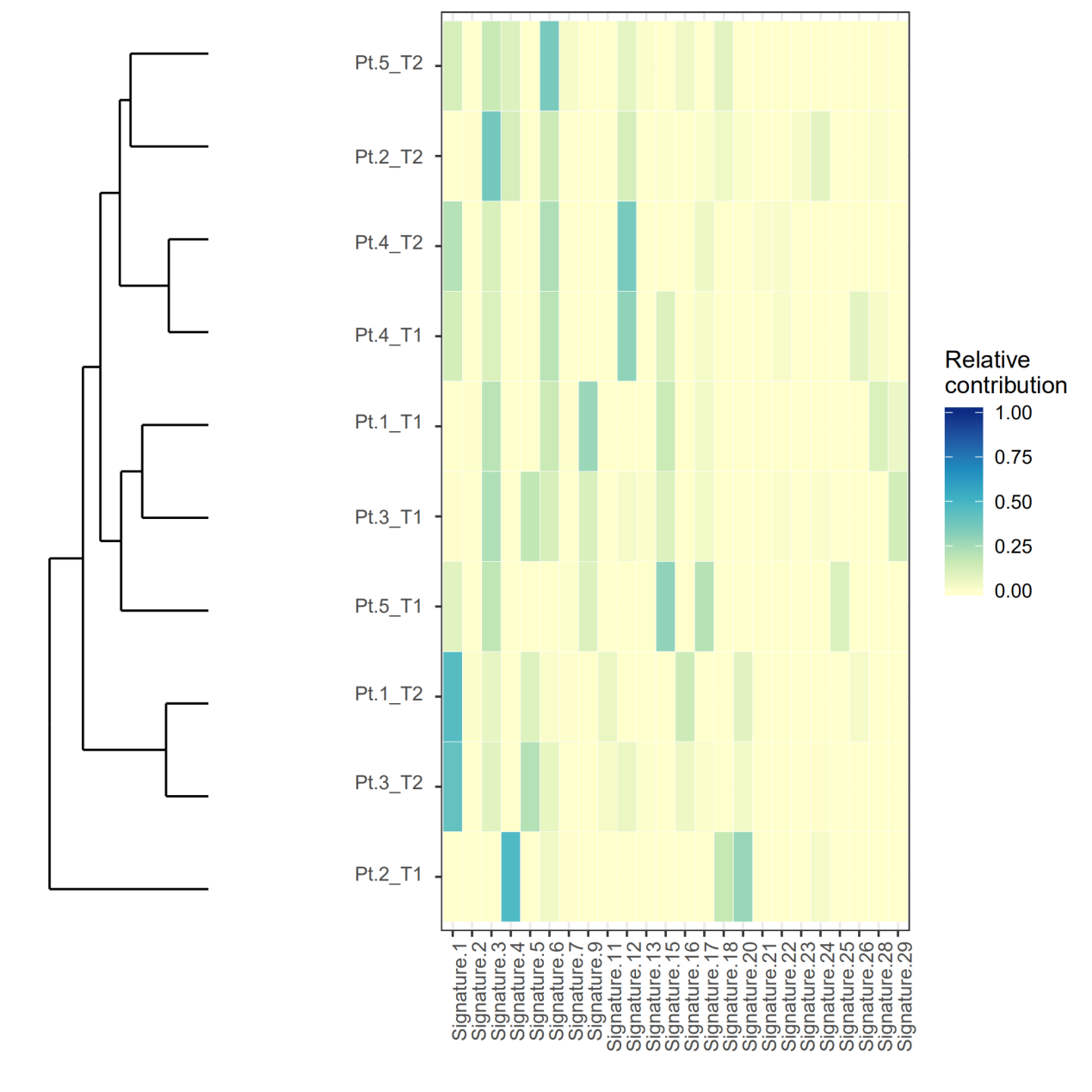
**

**Figure S1.** Cluster dendrogram showing the contribution of each composite mutation signatures to the overall mutation spectrum of each pre- and post-treatment sample. The increasing contribution is reflected in the shading from light yellow through to dark blue. The predominant signature is taken as the darkest blue shading for each sample.


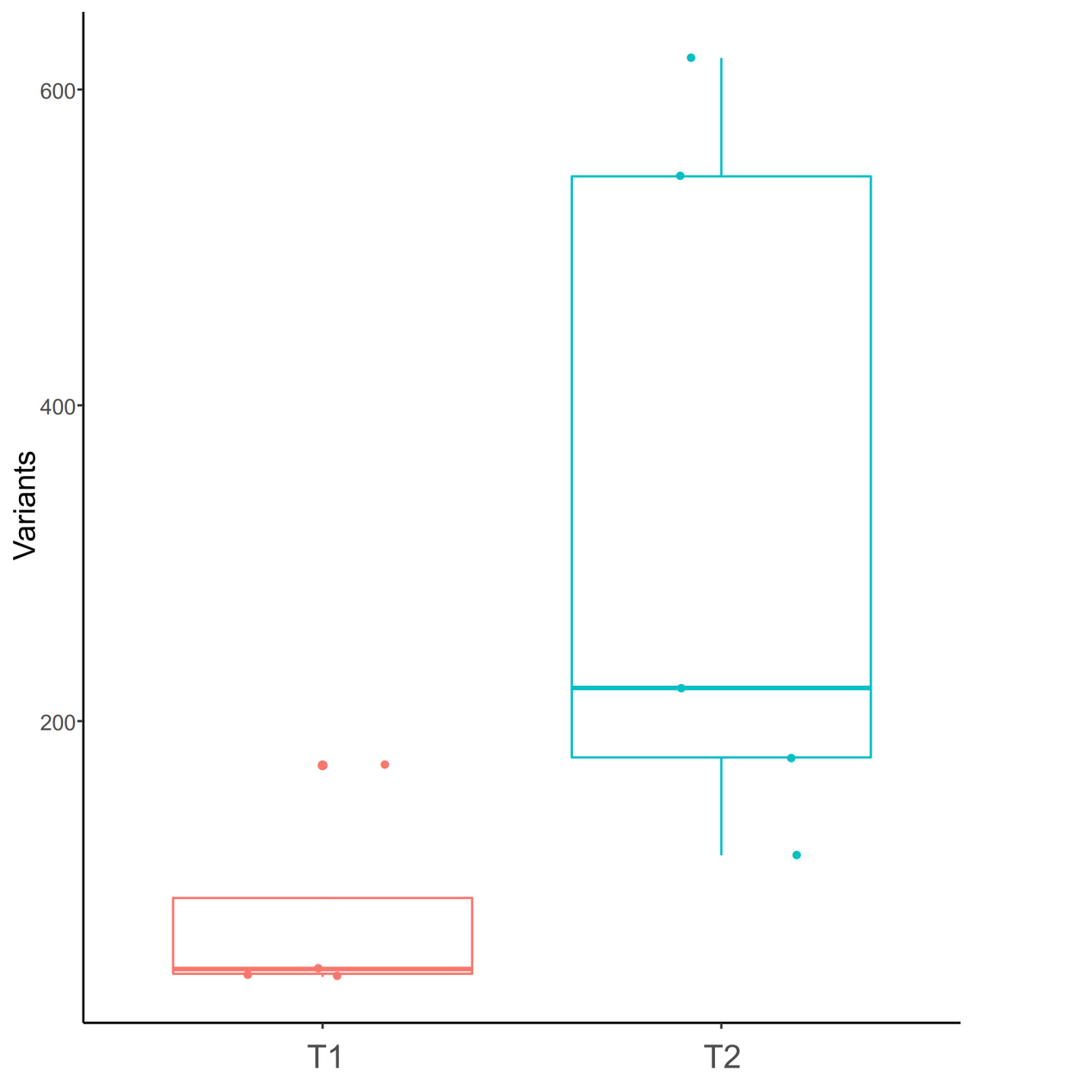


**Figure S2.** The number of mutations for pre-treatment samples (T2) and post-treatment samples (T1).


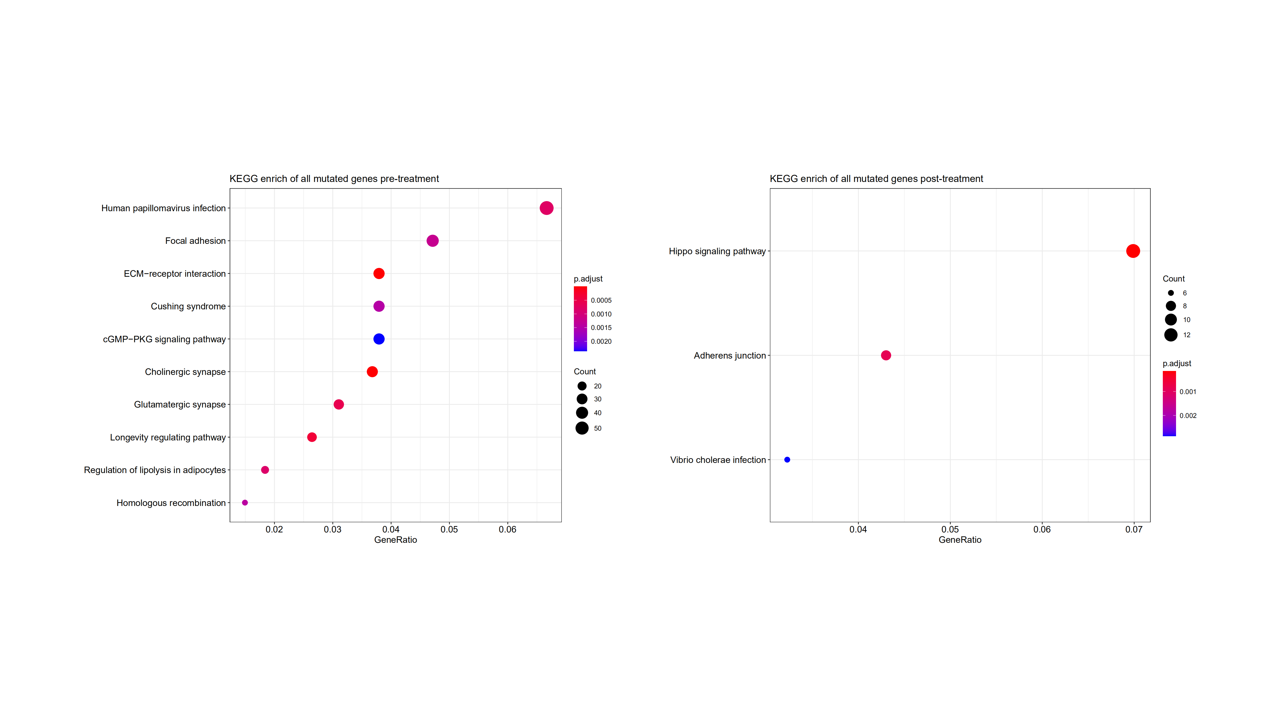


**Figure S3.** Kyoto Encyclopedia of Genes and Genomes (KEGG) enrichment analysis of all mutated genes for pre-treatment samples (T2) and post-treatment samples (T1).


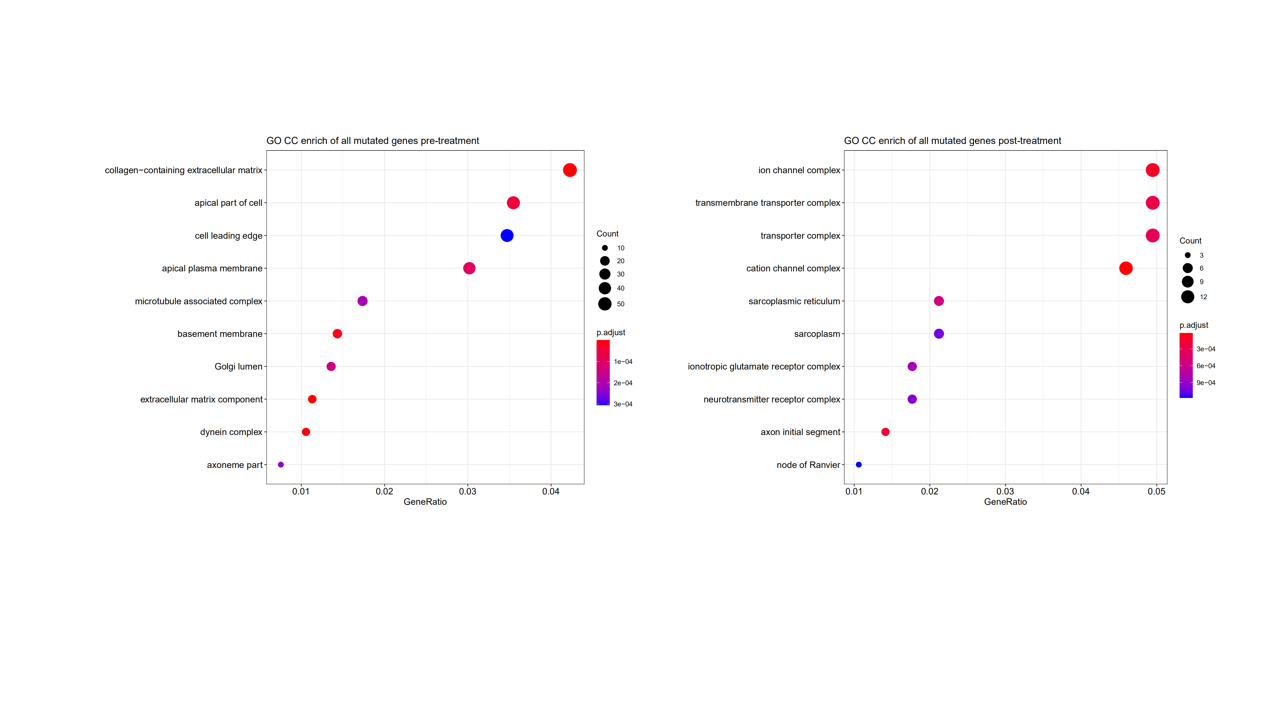


**Figure S4.** Gene Ontology (GO) cellular component (CC) enrichment analysis of all mutated genes for pre-treatment samples (T2) and post-treatment samples (T1).


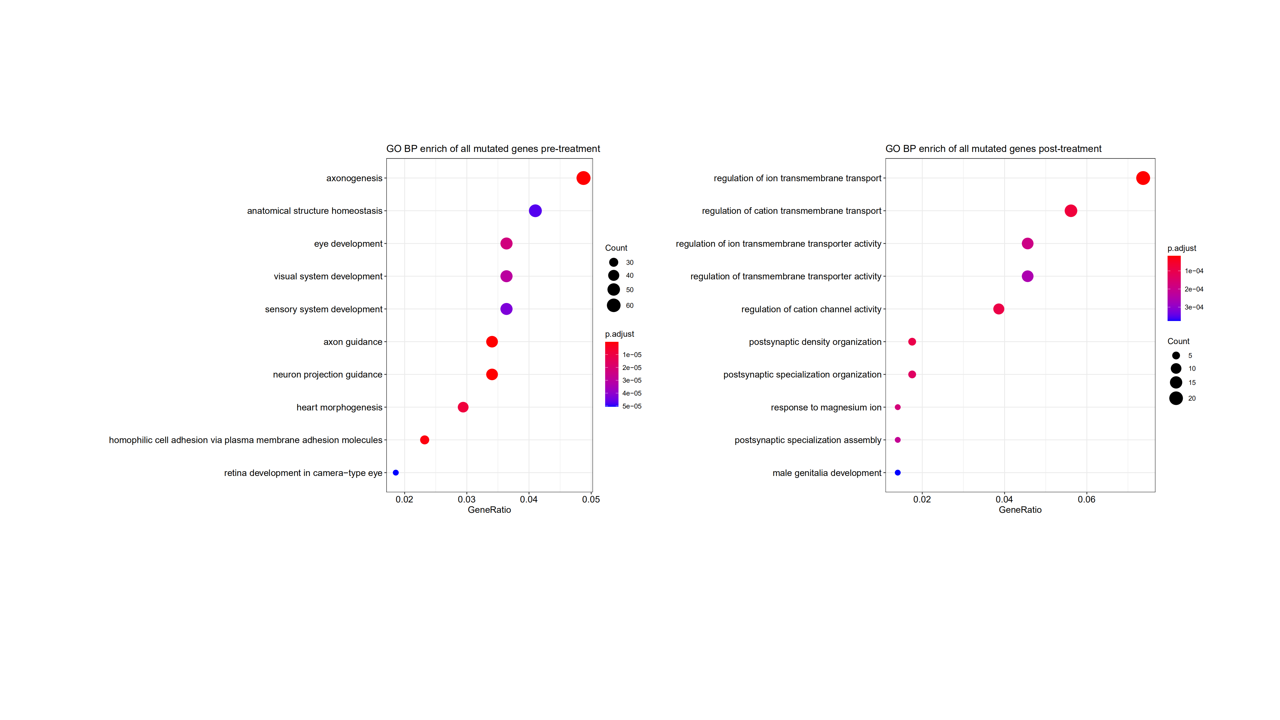


**Figure S5.** Gene Ontology (GO) biological process (BP) enrichment analysis of all mutated genes for pre-treatment samples (T2) and post-treatment samples (T1).
